# Trafficking of the exported *P. falciparum* chaperone PfHsp70x

**DOI:** 10.1038/srep36174

**Published:** 2016-11-08

**Authors:** Manuel Rhiel, Verena Bittl, Anke Tribensky, Sarah C. Charnaud, Maja Strecker, Sebastian Müller, Michael Lanzer, Cecilia Sanchez, Christine Schaeffer-Reiss, Benoit Westermann, Brendan S. Crabb, Paul R. Gilson, Simone Külzer, Jude M. Przyborski

**Affiliations:** 1 Parasitology, FB Biology, Philipps University Marburg, Karl von Frisch Strasse 8, 35043 Marburg, Germany; 2 Biochemistry Center (BZH), University of Heidelberg, Im Neuenheimer Feld 328, 69120 Heidelberg, Germany; 3 Burnet Institute, Melbourne, Vic. 3004, Australia; 4 Monash University, Melbourne, Vic. 3800, Australia; 5 Zentrum für Infektiologie, Parasitologie, Im Neuenheimer Feld 324, 69120 Heidelberg, Germany; 6 Laboratoire de Spectrométrie de Masse BioOrganique (LSMBO), IPHC, Université de Strasbourg, CNRS, UMR 7178, Strasbourg, France; 7 University of Melbourne, Melbourne, Vic. 3010, Australia; 8 Research School of Biology, ANU, Acton, ACT 2601, Australia

## Abstract

*Plasmodium falciparum* extensively modifies its chosen host cell, the mature human erythrocyte. This remodelling is carried out by parasite-encoded proteins that are exported into the host cell. To gain access to the human red blood cell, these proteins must cross the parasitophorous vacuole, a membrane bound compartment surrounding the parasite that is generated during the invasion process. Many exported proteins carry a so-called PEXEL/HT signal that directs their transport. We recently reported the unexpected finding of a species-restricted parasite-encoded Hsp70, termed PfHsp70x, which is exported into the host erythrocyte cytosol. PfHsp70x lacks a classical PEXEL/HT motif, and its transport appears to be mediated by a 7 amino acid motif directly following the hydrophobic N-terminal secretory signal. In this report, we analyse this short targeting sequence in detail. Surprisingly, both a reversed and scrambled version of the motif retained the capacity to confer protein export. Site directed mutagenesis of glutamate residues within this region leads to a block of protein trafficking within the lumen of the PV. In contrast to PEXEL-containing proteins, the targeting signal is not cleaved, but appears to be acetylated. Furthermore we show that, like other exported proteins, trafficking of PfHsp70x requires the vacuolar translocon, PTEX.

*Plasmodium falciparum* is responsible for the most serious form of human malaria, which causes over 0.6 million deaths annually. Most of these deaths occur in sub-Saharan Africa in children under the age of 5^1^. The pathology associated with *P. falciparum* infection is largely due to the capacity of parasite-infected human erythrocytes (the chosen host cell) to attach to various receptors on endothelial tissue within the body^2^. This phenomenon is dependent on the ability of the parasite to remodel its host cell. Remodelling is believed to be carried out by parasite-manufactured proteins that are trafficked out into the erythrocyte compartment^3^,^4^,^5^. As the erythrocyte itself is unable to traffic proteins, this unusual process is performed by parasite effectors and has become the subject of much research interest in recent years, as interrupting these pathways may provide a novel intervention strategy in the fight against malaria^6^.

A major breakthrough in our understanding of protein export in malaria parasites came with the discovery that many exported parasite proteins contain an N-terminal conserved trafficking signal, referred to as PEXEL or HT^4^,^5^,^7^. Mutation of this motif leads to a block in protein transport to the host cell. Subsequently it was established that this motif was a cleavage site for the aspartyl protease Plasmepsin V, and that cleavage was required for efficient protein export^8^,^9^,^10^,^11^. PEXEL proteins first enter the secretory pathway at the endoplasmic reticulum (ER), directed by an N-terminal secretory signal sequence (Fig. 1A,I). Within the ER their PEXEL/HT motif is cleaved by Plasmepsin V after which they continue along the secretory pathway before finally being released into the lumen of the parasitophorous vacuole (PV) where they undergo unfolding, possibly facilitated by molecular chaperones (Fig. 1A,II). Following unfolding, these proteins are believed to then pass through a PVM spanning protein translocon, PTEX^12^,^13^, before emerging into the erythrocyte cytosol where they refold, possibly also with the aid of further molecular chaperones (Fig. 1A,III) (for a thorough discussion on protein trafficking in *P. falciparum*, please see ref. 14).

Not all exported proteins however contain a PEXEL/HT motif. These so-called PEXEL-negative exported proteins (PNEPs) seem to rely on different trafficking motifs, some of which appear highly cryptic and require the inclusion of both amino acid motifs and structural elements including a putative trans-membrane domain^15^. Recent results suggest that the N-terminus of trans-membrane containing PNEPs may have similarities to the processed N-terminus of PEXEL proteins and may guide the protein into a similar export pathway^16^. As noted, most PNEPs reported so far contain a hydrophobic putative trans-membrane domain, however the ring exported protein 1 (REX1) appears to lack an internal hydrophobic domain and its transport is mediated by an N-terminal hydrophobic secretory signal sequence and a stretch of downstream amino acids^17^. The importance of individual amino acids in this export motif has however not been elucidated. Heiber *et al.* have also reported the identification of two further PNEPs that appear to contain N-terminal ER-type signal sequences^18^ but exact motifs driving transport of these proteins also remain unknown.

We have previously identified an exported Hsp70 (PfHsp70x) in *P. falciparum*-infected erythrocytes^19^. This protein contains an N-terminal secretory signal sequence, lacks a PEXEL/HT signal, but is nevertheless partially exported to the host erythrocyte and can thus be regarded as a PNEP. Trafficking of PfHsp70x to the host cell appears to be driven by the 32 N-terminal amino acids, including the signal sequence, upstream of the highly conserved Hsp70 ATPase domain (Fig. 1B)^19^. After removal of the signal sequence at its predicted cleavage site (HT-AS) the ATPase domain remains appended with 7 amino acids which we envision may be responsible for its trafficking into the erythrocyte compartment^19^. To further understand the role of this so called Hsp70x export motif, and if it could also serve as a model for soluble PNEPs, we have generated transgenic parasites expressing a GFP reporter fused to mutated versions of the Hsp70x export motif. Surprisingly, reversing the order of the amino acids in this region did not lead to a block in protein export, nor did scrambling the order of the residues. To understand this phenomenon we then carried out a site-directed mutagenesis of individual residues within this motif. We found that replacing glutamate residues within this region with alanine leads to a block in protein export. Unexpectedly, exchanging the acidic glutamate residues for acidic aspartic acids did not enable protein export, suggesting that trafficking is not directed by the acidic nature of the motif alone, but may rely on subtler biochemical properties of the motif’s amino acids. To study whether PfHsp70x export takes place via the PTEX translocon, we studied export in two conditional PTEX knockdown parasite lines. Indeed, export of PfHsp70x was reduced upon downregulation of PTEX150, or inactivation of PfHsp101 (both components of the translocon), suggesting that this translocon can accept not only PEXEL proteins, but also soluble PNEPs such as PfHsp70x as substrates. Finally, we demonstrate for the first time that that the mature N-terminus of PfHsp70x is acetylated, which may be involved in directing it’s trafficking. Our study represents the first to finely decipher the trafficking information necessary for export of a soluble PNEP to the *P. falciparum*-infected human erythrocyte and to elucidate the export pathway.

## Results

### GFP does not act as a spacer to increase export efficiency

**Figure 2:**
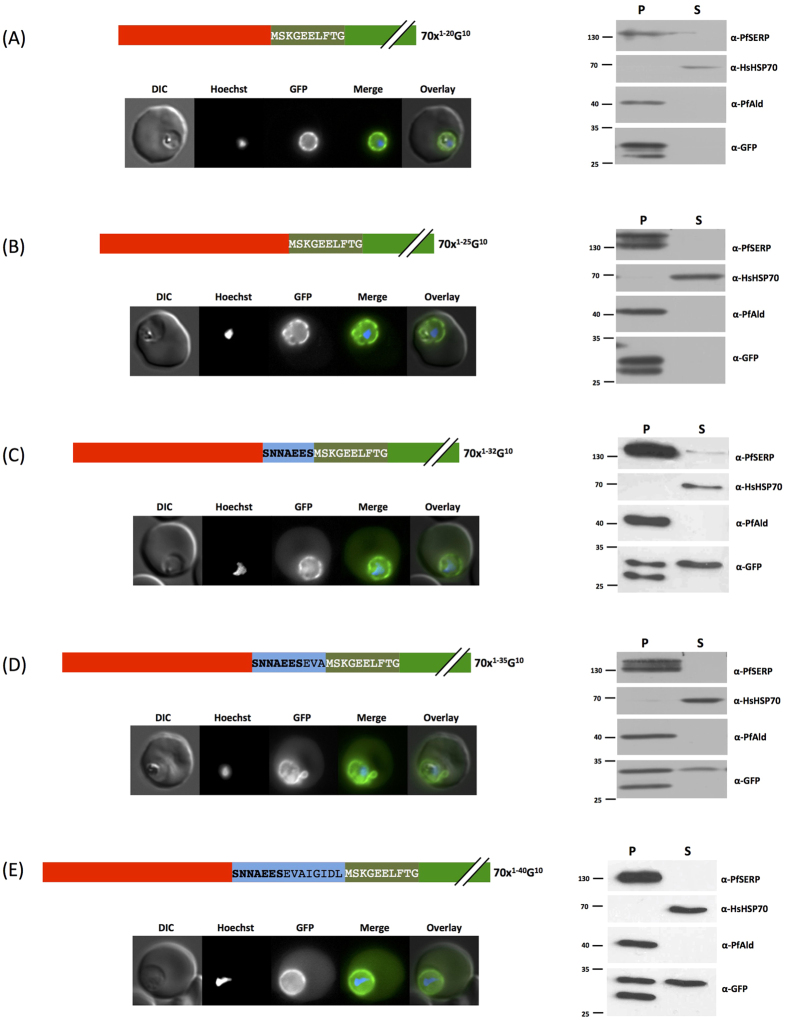
Influence of a GFP-derived linker on trafficking. Left panel shows structure of expressed PfHsp70x-GFP chimeras and live cell imaging of transfectants, right shows Streptolysin O fractionation followed by western blot using the antibodies indicated. DIC, differential interference contrast; in merge and overlay blue, Hoechst (nuclear stain); green, GFP; S, supernatant following SLO fractionation; P, pellet following SLO fractionation. Size markers in kDa. Pictures are representative of at least 10 individual observations, western blots of at least 3 independent experiments. The lower GFP band in the pellet fraction is a commonly observed GFP degradation product.

We have previously shown that a reporter protein containing the first 32 N-terminal amino acids of PfHsp70x fused to GFP is partially exported to the host cell cytosol, mirroring the distribution of the endogenous full-length protein^19^. Earlier studies have revealed that the presence of a GFP reporter directly adjacent to potential trafficking motifs may either inhibit or be conducive to protein transport^20^,^21^. We wished to investigate whether the GFP reporter itself may be acting as a spacer which may either increase or decrease export efficiency. For this reason we generated parasites expressing the first 20, 25, 32 and 35 and 40 N-terminal amino acids of PfHsp70x, but now including a spacer consisting of the first 10 amino acids of the GFP reporter in front of the actual full-length GFP. In all cases, distribution of the GFP chimera was similar to that of the wild type reporters (Fig. 2A–E)^19^, with fluorescence being detected both in the PV and erythrocyte. We verified our fluorescence microscopy using streptolysin O (SLO) fractionation. SLO is a bacterial pore-forming protein which permeabilises the erythrocyte plasma membrane, while leaving the PV intact. Treatment with SLO thus allows separation of infected erythrocytes into supernatant (erythrocyte cytosol) and pellet (PV and parasite cytosol) fractions^22^. To control the fractionation, we also detected markers for specific compartments: HsHsp70, host cell cytosol; PfSERP, PV lumen; PfAldolase, parasite cytosol. In all cases the control proteins were consistently found in the correct fraction, whilst only the chimera including more than 32 N-terminal amino acids of PfHsp70x could be found in the exported fraction (Fig. 2A–E), corroborating our observations using fluorescence microscopy.

This result demonstrates that the 7 amino acid motif is a *bona fide* isolated trafficking motif and is not just required as a steric linker between the signal peptide and folded GFP.

### Reversal or scrambling of the trafficking motif has no effect on protein export efficienc**y**

**Figure 3:**
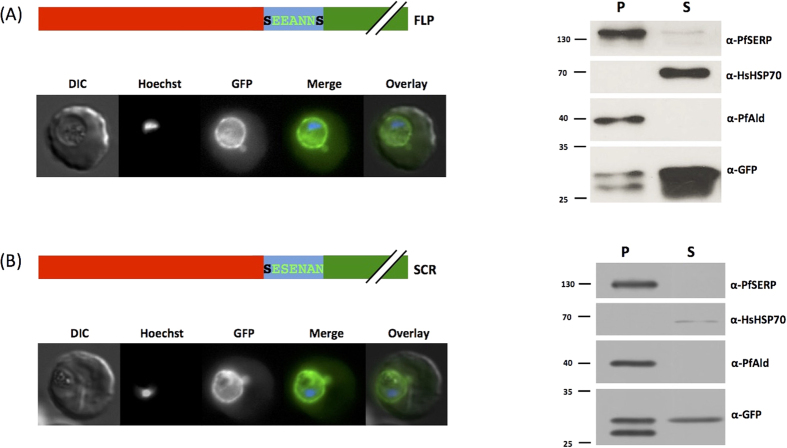
Reversal and motif scrambling has no effect on export. Left panel shows structure of expressed chimera and live cell imaging of transfectants, right shows Streptolysin O fractionation followed by western blot using the antibodies indicated. Changes to motif due to reversal or scrambling are shown in light green, original motif in bold. Red indicates the first 25 amino acids of PfHsp70x, predicted signal sequence. DIC, differential interference contrast; in merge and overlay blue, Hoechst (nuclear stain); green, GFP; S, supernatant following SLO fractionation; P, pellet following SLO fractionation. Size markers in kDa. Pictures are representative of at least 10 individual observations, western blots of at least 3 independent experiments. The lower GFP band in the pellet fraction is a commonly observed GFP degradation product.

Having demonstrated that the 7 amino acid motif alone is responsible for the observed export, we next investigated the effect of reversing the order of the amino acids. A block in transport caused by this would indicate a potential role for a specific receptor in the export process. We therefore engineered parasites to express the first 25 N-terminal amino acids of PfHsp70x (the signal sequence) fused to a “flipped” version of the export motif (SNNAEES > SEEANNS, the first and final positions of the motif remain constant due to their position in the wild-type sequence, referred to as FLP). To our surprise, in parasites expressing this chimera, fluorescence could still be observed in the erythrocyte cytosol (Fig. 3A, left panel). Sub-cellular fractionation also revealed a portion of GFP in the erythrocyte cytosol (Fig. 3A, right panel). This unexpected result led us to further alter the order of the amino acids in the motif. We scrambled the sequence (SNNAEES > SESENAN, the first position remained the same by chance, referred to as SCR) and generated further transfectant parasite lines. Once again, despite the order of the amino acid in the motif being different to the wild-type, GFP could be observed in the erythrocyte cytosol by fluorescence microscopy (Fig. 3B, left panel) and sub-cellular fractionation (Fig. 3B, right panel).

### Site directed mutagenesis of the motif identifies residues essential for export

**Figure 4:**
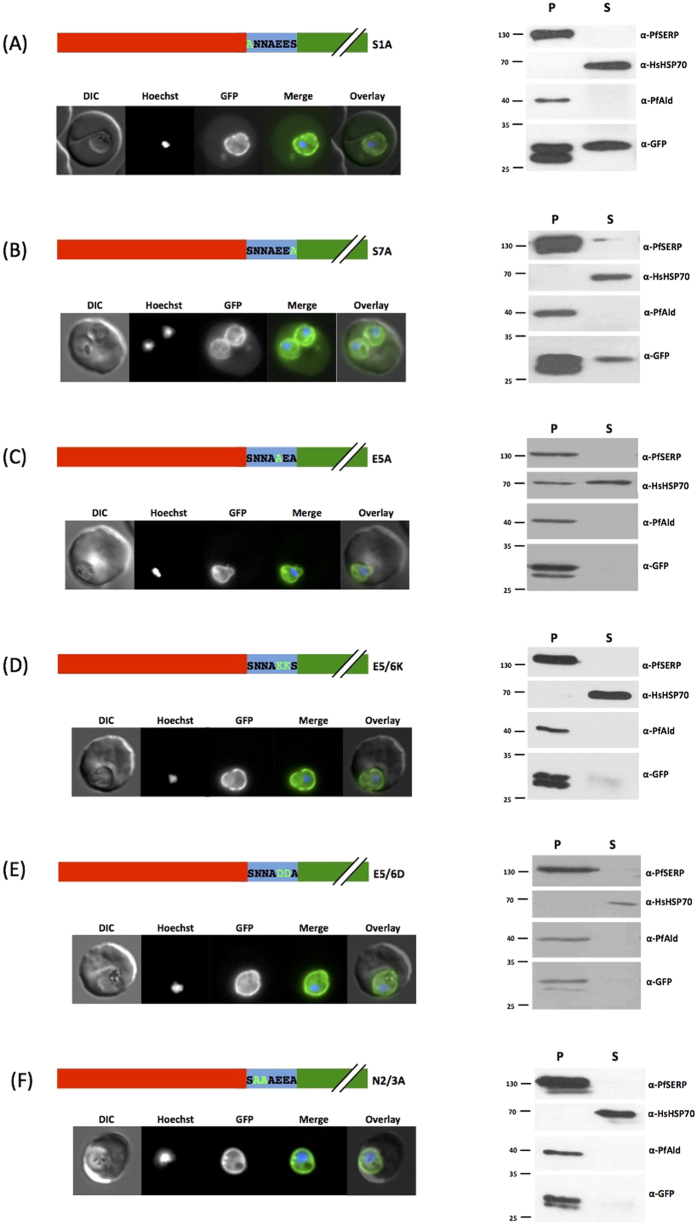
Site directed mutagenesis of the export motif. Left panel shows structure of expressed PfHsp70x-GFP chimeras and live cell imaging of transfectants, right shows Streptolysin O fractionation followed by western blot using the antibodies indicated. Mutated residues are shown in light green, original motif in bold. Red indicates the first 25 amino acids of PfHsp70x, predicted signal sequence. Numbering refers to position in motif following signal sequence cleavage. DIC, differential interference contrast; in merge and overlay blue, Hoechst (nuclear stain); green, GFP; S, supernatant following SLO fractionation; P, pellet following SLO fractionation. Size markers in kDa. Pictures are representative of at least 10 individual observations, western blots of at least 3 independent experiments. The lower GFP band in the pellet fraction is a commonly observed GFP degradation product.

As the order of the residues did not seem to have a major effect on protein export, we started a systematic mutagenesis of the individual residues within the export motif. As serine residues can potentially be phosphorylated, and thus may act as an export signal, we mutated each individual serine in the motif (at position 1 and 7) to an alanine (S1A, S7A). In neither case did we observe a reduction in protein export, with GFP still seen in the erythrocyte by both fluorescence microscopy and sub-cellular fractionation (Fig. 4A,B, left and right panels). The motif contains two acidic glutamate residues at positions 5 and 6. Mutation of E5 to alanine (E5A) resulted in a block of reporter protein export with GFP only being detected associated with the parasite, likely representing the lumen of the PV (Fig. 4C, left and right panels). Similarly, replacement of glutamate with the basic amino acid lysine (E5/6K) resulted in no protein export (Fig. 4D, left and right panels). As these results suggested a possible role for negative charge in the functional export motif, we swapped the negatively charged glutamate residues for similarly negative aspartate (E5/6D). This could however not restore export, with no GFP being detected in erythrocyte cytosolic fractions (Fig. 4E, left and right panels). Finally, we mutated the two asparagine residues in positions 2 and 3 for alanine (N2/3A). Again, this caused a block in protein traffic to the host cell (Fig. 4F, left and right panels).

**Figure 5:**
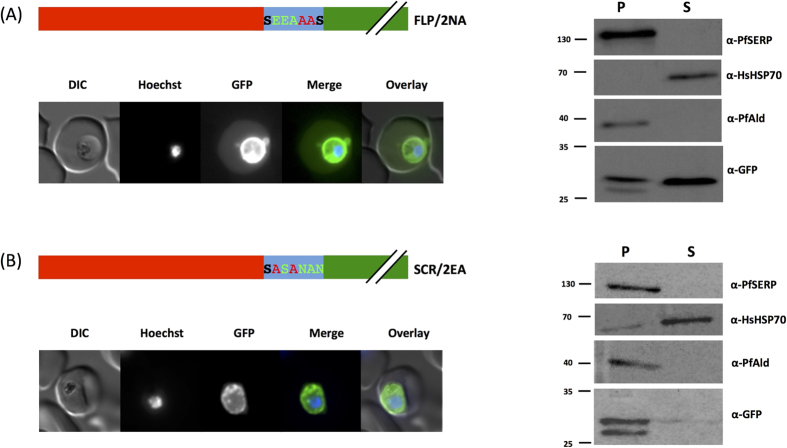
Introduction of mutations into SCR and FLP constructs. Left panel shows structure of expressed chimera and live cell imaging of transfectants, right shows Streptolysin O fractionation followed by western blot using the antibodies indicated. Residues of the original SCR and FLP motifs are shown in light green, introduced mutations in red, residues remaining in the same position as the wild-type motif in bold. Red indicates the first 25 amino acids of PfHsp70x, predicted signal sequence. DIC, differential interference contrast; in merge and overlay blue, Hoechst (nuclear stain); green, GFP; S, supernatant following SLO fractionation; P, pellet following SLO fractionation. Size markers in kDa. Pictures are representative of at least 10 individual observations, western blots of at least 3 independent experiments. The lower GFP band in the pellet fraction is a commonly observed GFP degradation product.

*In silico* analysis of the asparagine mutants suggested that the signal peptidase cleavage site might have shifted to an atypical site due to the mutation, removing part of the trafficking motif. For this reason we decided to introduce the same mutation into the FLP background sequence, and study the effect on protein traffic. Indeed, this construct was trafficked to the host cell (Fig. 5A), suggesting that the lack of export of the asparagine mutant in the wild type background is due to aberrant signal sequence cleavage, possibly disturbing correct recognition of the export motif, or causing removal by signal peptidase.

Finally, to verify that only the glutamate residues are required for export, independent of the surrounding sequence context, we mutated the glutamate residues in the SCR background to alanine. These mutations blocked traffic of the reporter protein to the host cell (Fig. 5B), verifying the essential role of the glutamate residues in transport across the PV. All results were also verified by sub-cellular fractionation (Figs 4A–F and 5A,B).

### The signal sequence of PfHsp70x contains no protein-specific targeting information and trafficking of PfHsp70x is sensitive to BFA

**Figure 6:**
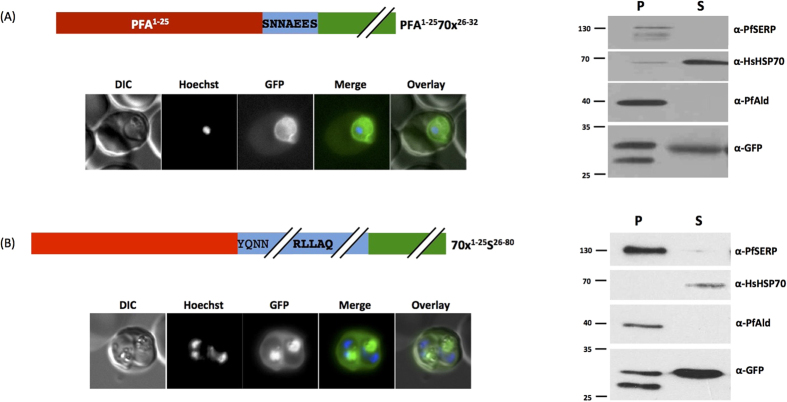
The nature of the signal sequence does not influence protein export. Left panel shows structure of expressed chimera and live cell imaging of transfectants, right shows Streptolysin O fractionation followed by western blot using the antibodies indicated. (**A**) Light red indicates predicted signal sequence from PfHsp70x, dark red from the exported co-chaperone PFA660 (PF3D7_0113700). In (**A**) PfHsp70x export motif is shown in bold. In (**B**) PEXEL motif is shown in bold. DIC, differential interference contrast; in merge and overlay blue, Hoechst (nuclear stain); green, GFP; S, supernatant following SLO fractionation; P, pellet following SLO fractionation. Size markers in kDa. Pictures are representative of at least 10 individual observations, western blots of at least 3 independent experiments. The lower GFP band in the pellet fraction is a commonly observed GFP degradation product.

Having isolated the specific residues required for the function of the export sequence, we wished to investigate whether the predicted hydrophobic N-terminal signal sequence of PfHsp70x may be involved in directing the chaperone to a specific protein export pathway, possibly at the level of the ER. For this reason we generated two new transfectant lines. We fused the predicted signal sequence from a non-related protein (PFA660, an exported Hsp40 localised to the J-dots in infected cells^23^) to the 7 amino acid motif from PfHsp70x. Transfectants expressing this construct showed fluorescence in the host cell cytosol, but also in the PV, similar to the wild-type PfHsp70x sequence (Fig. 6A, left panel). Conversely, we fused the predicted signal sequence from PfHsp70x to amino acids 23–80 derived from the STEVOR protein. This protein is exported by the classical PEXEL/HT pathway, and contains a canonical PEXEL sequence at amino acid position 48–52^7^. In this case fluorescence could be observed within the erythrocyte cytosol and also in the food vacuole of intracellular parasites, similar to previous published results using the first 80 amino acids of STEVOR (Fig. 6B, left panel)^7^. The food vacuolar GFP is the result of internalization of host cytosol during the parasites feeding activities. No GFP could be detected within the PV, suggesting that protein export was highly efficient (Fig. 6B, left panel). These results were all verified by sub-cellular fractionation (Fig. 6A,B, right panels). This data suggests that the choice of N-terminal signal sequence had no effect on eventual protein sub-cellular localisation, and that the 7 amino acid sequence found in PfHsp70x contains all the information required for protein translocation to the host cell.

**Figure 7:**
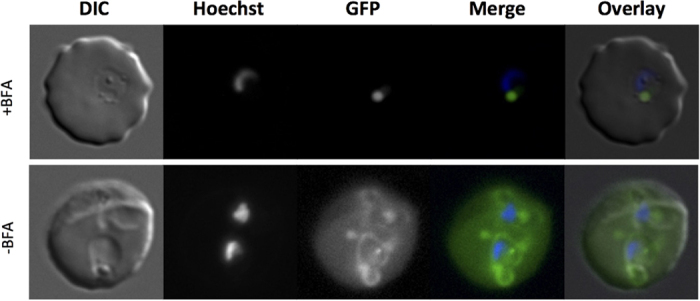
BFA blocks protein secretion and export. Live cell imaging of transfectants expressing PfHsp70x^1-40^-GFP+/− BFA. In merge and overlay blue, Hoechst (nuclear stain); green, GFP. Pictures are representative of at least 10 individual observations. BFA, Brefeldin A.

**Figure 8:**
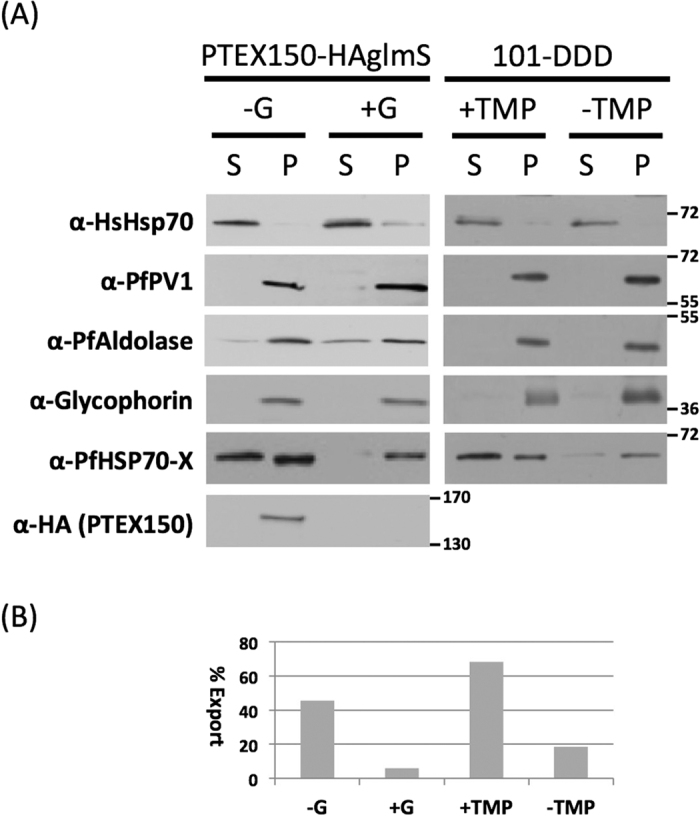
The vacuolar translocon PTEX is required for export of PfHsp70x. (**A**) The distribution of PfHsp70x in tetanolysin supernatant and pellet was detected following treatment to inactivate PTEX. G, glucosamine; TMP, trimethoprim; S, supernatant following tetanolysin fractionation; P, pellet following tetanolysin fractionation. Size markers in kDa. Blots shown are representative of 3 independent experiments. (**B**) Quantification of data shown in (**A**).

Although the presence of an N-terminal hydrophobic signal sequence suggests that PfHsp70x initially co-translationally enters the ER, we nevertheless wished to investigate the influence of brefeldin A (BFA^24^) on transport, to exclude sequestration of PfHsp70x into an alternative secretory pathway possibly specialized in exported proteins. BFA is a fungal metabolite that blocks canonical secretory traffic by inducing retrograde movement of secretory vesicles from the Golgi to the ER. For this reason we treated highly synchronized ring-stage transgenic parasites expressing our Hsp70x^1-40^-GFP chimera with 5 μg/ml BFA for 16 hours. Following treatment, we then observed the distribution of the GFP reporter (Fig. 7). Parasites treated with BFA were arrested in the ring stage, whereas non-treated parasites progressed to early trophozoite stage (Fig. 7). As expected, in non-treated parasites, fluorescence could be observed in the host cell cytosol and in a ring structure surrounding the body of the parasite, representing the parasitophorous vacuole (Fig. 8, lower panel). In treated cultures, GFP could only be observed in a peri-nuclear dot structure. This structure has previously been shown to represent the ER/Golgi structure following BFA treatment^7^,^25^. These results convincingly demonstrate that trafficking of PfHsp70x takes place initially via the default secretory pathway.

### Transport of PfHsp70x to the host cell requires an active PTEX translocon complex

Recent studies have suggested that export of both PEXEL, and PNE proteins takes place via the PTEX translocon^13^,^26^. To investigate whether PfHsp70x is also exported in this manner, we studied export of PfHsp70x in cell lines in which levels of the essential PTEX component PTEX150 can be downregulated using a glmS riboswitch system^13^, or in which activity of a further component, PfHsp101, can be conditionally inhibited^26^. Synchronized parasite cultures at 20–24 hours post invasion containing either the PTEX150-HA*glmS* or Hsp101^DDD^ were left untreated, or grown in the presence of 1mM glucosamine (*glmS* line) or 10 μM TMP until 16 hours post invasion in the next cycle. Infected erythrocytes were then permeabilised with tetanolysin (which lyses only the erythrocyte plasma membrane, releasing exported proteins) and separated into a supernatant and pellet fraction. We then analysed both fractions by western blot. To verify the fractionation procedure, we detected HsHsp70 (a marker of the host cell cytosol), PfPV1 (a marker of the PV lumen) and PfAldolase (a marker of the parasite cytosol). All three markers were found only in the expected fraction (Fig. 8A). In the PTEX150-HA*glmS* cell line grown in the presence of 1 mM glucosamine, expression of PTEX150 was greatly reduced when compared to non-treated cells (Fig. 8A). Western blotting revealed that glucosamine treatment also led to a major reduction in export of PfHsp70x to the host cell cytosol compared to the non-treated control (6% vs 45%, Fig. 8A,B). Similarly, Hsp101^DDD^ cells grown in the absence of TMP (thus inhibiting function of PfHsp101) exhibited reduced export of PfHsp70x compared to the control (18% vs 68%, Fig. 8A,B). As a control for the inactivation of PTEX, we analysed export of the PEXEL protein KAHRP, and the PNEP SBP1 by immunofluorescence on fixed cells (Supplementary Fig. S1). As previously reported, in both cell lines, inactivation of the PTEX translocon lead to a block of protein export and an accumulation of the protein around the parasite itself^13^,^26^. These data support a role for both PTEX150 and PfHsp101, and thus the vacuolar translocon PTEX, in translocation of PfHsp70x to the host cell cytosol.

### The trafficking motif is not cleaved, but the mature N-terminus of PfHsp70x is acetylated

**Figure 9:**
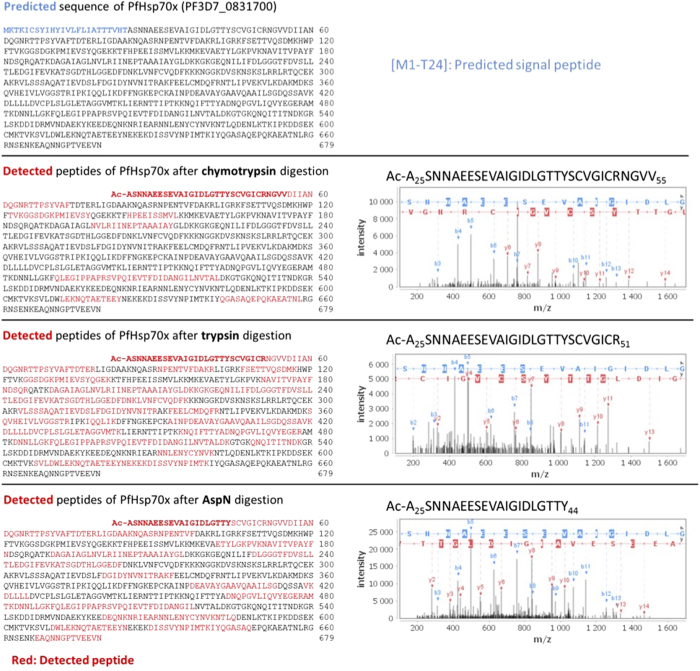
The trafficking motif is not cleaved but acetylated. Identified peptides of PfHsp70x after chymotrypsin, trypsin and AspN digestion respectively. On the left panel, in red are highlighted the sequence coverage obtained for each digestion, in bold red the N-terminal peptide. In the right panel, the MS/MS spectra of acetylated N-terminal peptides of PfHsp70x.

It is generally accepted that PEXEL acts as a recognition sequence for the ER protease Plasmepsin V (PMV), and is cleaved and acetylated, leaving a new N-terminus with the structure Ac-xE/D/Q^8^,^9^,^10^. Additionally, it has previously been reported that the N-terminus of the PNEP REX2 is cleaved, however it is unknown whether this is a general phenomenon for PNEPs, and whether such cleavage is required for protein export^27^. We were thus interested in identifying the mature N-terminus of PfHsp70x following signal peptide cleavage. To this end, we carried out an N-terminome analysis of infected red blood cells using the doublet N-terminal oriented (dN-TOP) approach^28^. No peptides corresponding to the predicted signal sequence (as is expected if signal peptide removal takes place co-translationally) were identified in none of the three different experiments (trypsin, chymotrypsin and AspN digestions). However, we were able to identify peptides starting after the predicted signal peptide cleavage site (HT-AS), and containing the identified export motif (SNNAEES, Fig. 9). These peptides additionally were N-acetylated, irrespective of the protease used for the initial digest.

## Discussion

Since the discovery that malaria parasites synthesise and traffic their own proteins to and through the infected host-erythrocyte, much research effort has been focused on understanding this unusual novel protein trafficking system, including analysis of the signals, mechanisms and machinery required for high-fidelity delivery of proteins to specific sub-cellular compartments. These studies have revealed that protein export appears to be a signal-driven process which furthermore requires the presence of a number of parasite derived molecules for maximal effectiveness^3^,^4^,^5^,^29^. One molecule which is predicted to play an important role in protein traffic through the erythrocyte is the exported chaperone PfHsp70x, which is dually localised to the lumen of the PV and intra-erythrocytic structures named J-dots^19^,^23^. This protein does not contain any previously characterised protein export signal, but transport to the erythrocyte appears to be directed by a 7 amino acid motif found directly following a hydrophobic signal sequence^19^. Here, through expression of GFP chimera, we have investigated this motif in detail. Our initial results were surprising as they showed that reversal of the amino acid motif (FLP) did not ablate protein transport, nor did scrambling the order of the amino acids (SCR). This suggests that a purely structural motif is unlikely to be the recognition signal for protein export. We noticed that, in both the FLP and SCR mutants, negatively charged residues are still found in close proximity to each other. Removal of the negative charge by replacement of glutamate for alanine or charge reversal by replacement with lysine led to a block in protein export. However, this effect could not be complimented by reintroducing the negative charge with aspartate, suggesting that negative charge itself is not the only defining factor for export. Removal of serine residues did not block protein export, excluding a potential role for post-translational modifications such as serine phosphorylation. Exchange of asparagine residues for alanine in the wild-type sequence also resulted in a block in protein export, however this is likely to be a side effect of an incorrect signal sequence cleavage which may remove part of the export sequence, as mutation of these residues in the FLP background did not block protein export.

The aforementioned PEXEL/HT motif functions as a recognition site for a specific protease, Plasmepsin V. Although not fully understood in mechanistic detail, cleavage by Plasmepsin V seems to direct the substrate protein into an export pathway^8^,^10^, possibly already in the ER. The cleaved PEXEL also appears to be acetylated, although the significance of this for export remains elusive. Whether N-terminal cleavage of PNEPs is required for export remains poorly understood, and such cleavage has only been demonstrated for a single protein^27^. Our data suggest that PfHsp70x does not undergo any further proteolytic processing following removal of the signal sequence, and thus this can be discounted as a prerequisite for trafficking to the host cell. However, the N-terminus of mature PfHsp70x is acetylated, possibly suggesting that the acetylation itself, rather than cleavage by a specific protease might be essential for protein export. Whether this is a general principle, or represents an isolated case of PNEP acetylation is unclear, and further studies will be required to elucidate the role of N-acetylation in export of both PEXEL and PNE proteins.

Although it is still unknown at a molecular level how exported proteins are actually recognised, recent studies suggest that both PEXEL and PNE proteins converge at the vacuolar translocon PTEX for transport across the PVM. Our data also support this view, as interfering with the function of PTEX blocks traffic of PfHsp70x to the erythrocyte cytosol. Given that the PTEX translocon complex must deal with a range of substrates exposing an array of different N-termini, we feel it unlikely that substrate recognition itself takes place directly at the translocon itself, but may occur at an earlier stage in the export pathway.

In this manuscript we have concentrated on identifying residues that are required for export of PfHsp70x to the host cell. However, in contrast to the situation for most PEXEL/HT containing proteins, even the full PfHsp70x export motif leads to only partial export of the protein, with a large amount of reporter being retained within the PV. This situation reflects the distribution of endogenous PfHsp70x meaning that the minimal motif contains enough information to lead to a dual-localisation of the protein^19^. To exclude that the N-terminal signal peptide may in some way direct entry to a specific PNEP export pathway, we exchanged the PfHsp70x signal sequence with one derived from a PEXEL/HT protein. Similarly, we replaced the endogenous signal sequence in a PEXEL-containing STEVOR reporter with that from PfHsp70x^7^. In both cases the reporter was found in a similar distribution to constructs containing the endogenous signal sequences, excluding an influence of the signal sequence on protein fate. Furthermore, using BFA we could exclude that PfHsp70x is secreted via an alternative secretory pathway. We are unsure how one motif can lead to a dual localization, but similar observations have been made in various biological systems, suggesting that it may reflect a general biological principle for targeting one gene product to more than one compartment. Parasites are often genomically reduced and this tactic removes the need for two copies of one gene and the associated metabolic burden, with one gene product being targeted to two different sub-cellular localizations^30^. The 130 kDa glycophorin binding protein (PfGBP130) is also targeted to both PV and host cell, but this protein contains a canonical PEXEL sequence, and reporters containing only the N-terminal targeting information are fully exported, suggesting that the mechanism of dual localisation is distinct from that of PfHsp70x^5^,^22^,^31^.

One possibility that we have not addressed is that the GFP chimera may have been co-exported to the host cell together with endogenous PfHsp70x. However, we feel that such a co-transport is unlikely. It has previously been shown that trafficking through the PTEX translocon requires substrates to be held in a translocation-competent state (likely fully or partially unfolded), which would also exclude trafficking of protein complexes^31^. As PfHsp70x appears to be transported to the host cell via PTEX (Fig. 8), we believe that such a “piggy-back” mechanism is unlikely to explain transport of the chimera to the host cell.

The herein identified motif is only 7 amino acids long, and only 2 of the residues contained within appear essential for protein export, but this minimal motif is nevertheless capable of directing the trafficking of a protein consisting of over 600 amino acids. To date, this is the shortest stand-alone export sequence thus found in the *P. falciparum* system. While an exact understanding of the molecular mechanisms underlying protein export in malaria parasites requires further experimentation, our results once again highlight the variety of protein export sequences in the *P. falciparum* system. Why the parasite needs so many different signals to target proteins to the host cell still remains unclear, but also emphasizes the importance of this process for intra-erythrocytic survival of this most important of human parasites.

## Materials and Methods

### Plasmid Construction

All linker constructs used in this study were derived from vectors that have been previously described^19^. All primers used for construct generation are listed in Supplementary Material S1. Constructs 70x^1-20^, 70x^1-25^, 70x^1-32^, 70x^1-35^, and 70x^1-40^, encoding differentially truncated N-termini of Hsp70x fused to GFP^19^ were expanded by short linker sequences encoding for the first ten amino acids of GFP (G^10^) in order to generate the constructs 70x^1-20^G^10^-70x^1-40^G^10^. To this end, the complementary oligonucleotide pair GFP1_10_oligo_F/GFP1_10_oligo_R were annealed. The resulting short double stranded sequences were cloned in frame between the sequences encoding the N-terminal amino acids of Hsp70x and the C-terminal GFP coding sequence into the plasmids 70x^1-20^-70x^1-40(19)^ using AvrII und KpnI restriction sites. Constructs encoding the native signal peptide of Hsp70x (amino acids 1-25) followed by an altered export sequence (amino acids SNNAEES between positions 26–32) were generated via PCR. The oligonucleotide Hsp70_X_F was used as forward primer for all PCR reactions with the plasmid 70x^1-32^ serving as template. The PCR fragments were cloned in frame into the pARL2-GFP vector^7^ in front of the GFP coding region by using the restriction sites XhoI and AvrII. All constructs, the used reverse primers and the resulting mutated export sequences are found in Supplementary data. The role of the native signal peptide of Hsp70x was investigated with the two constructs PFA^1-25^70x^26-32^ and 70x^1-25^S^26-80^. PFA^1-25^70x^26-32^ encodes a chimeric targeting sequence that is a combination of the PFA660w signal peptide (amino acids 1–25) followed by the Hsp70x export sequence (amino acids 26–32). The construct was generated via PCR using the primers Pfa660w_70x_X_F and Pfa660w_70x_A_R and a plasmid carrying the PFA660w coding sequence^23^ as template. In contrast, the construct 70x^1-25^S^26-80^ encodes a chimeric targeting sequence that is combined of the native signal peptide of Hsp70x (amino acids 1–25) and the PEXEL-type export motif of STEVOR (amino acids 23–80). The construct was generated by overlapping-extension PCR. First, the N-terminal (70x^1-25^) and the C-terminal (S^26-80^) portion were generated separately via PCR. For 70x^1-25^, primers Hsp70x_Xho_F and Sew_R_70x+STV were used with the construct 70x^1-32(19)^ serving as template. S^26-80^ was amplified from the plasmid STEVOR^1-80^ 7 using the primers Sew_F_70x+STV and stev^A80rev^. The two resulting PCR products were used as template for a final PCR reaction with Hsp70x_Xho_F and stev^A80rev^. The chimeric targeting sequence coding PCR products were cloned in frame into the pARL2-GFP vector^7^ in front of the GFP coding region via using the restriction sites XhoI and AvrII. FLP/2NA, SCR/2EA: PCR fragments containing the required mutations were generated by PCR using FLP or SCR constructs as template, Hsp70_X_F as forward primer and Flp_2NA_AII_R or Scr_2EA_AII_R as reverse primer, and subsequently cloned into suitably restricted pARL2-GFP as above. All constructs were verified by restriction digest and automated sequencing prior to transfection.

### SLO and tetanolysin lysis

Trophozoite-infected erythrocytes were enriched to a parasitemia of approximately 70% using Gelafundin flotation^32^ and subsequently subcellular fractionated using streptolysin O (SLO) or tetanolysin. In brief: 2x10^8^ RBCs were washed in 200 μl PBS and pelleted by centrifugation at 3000 rpm for 2 minutes. The resulting pellet was resuspended in 166 μl PBS and treated with 14 μl SLO/tetanolysin, (4 hemolytic units). The permeabilization reaction was allowed to proceed for 6 minutes at room temperature and was then separated into a soluble and a pellet fraction by centrifugation for 30 sec. The soluble fraction was directly mixed with sample buffer and boiled at 95 °C for 10 minutes. The pellet was washed three times with 500 μl PBS and then dissolved in sample buffer and boiled at 95 °C for 10 minutes. 1 × 10^7^ cell equivalents were analysed by 12% SDS-PAGE and semi-dry immunoblotting. Antibodies used: mouse anti-GFP (1:1000, Roche), mouse anti-HsHsp70 (1:500, Santa Cruz), rabbit anti-PfSERP (1:500), rabbit anti-PfAldolase (1:5000), rabbit anti-PfHsp70x (1:1000^19^), rabbit anti-PfPV1 (1:500^33^). Secondary antibodies anti-mouse/rabbit HRP were used at 1:2000 (DAKO).

### Parasite culture and transfection

Parasites were cultivated under standard conditions^34^. Transfection of ring stage parasites was carried out by electroporation with an approximate 100 μg of plasmid DNA, and transfectants were selected by, and maintained with, 2.5 nM of WR WR99210 (Jacobus Pharmaceuticals)^35^.

### Sample preparation for nanoLC-MS/MS analyses

The protocol used was carried out according to the original dN-TOP publication with slight modifications^36^. Proteins were loaded and electrophoresed on a 12% SDS-PAGE and stained overnight with colloidal Coomassie Brillant Blue (BioSafe coomassie stain, Bio-Rad). Whole lanes were manually cut in bands of 2 mm, proteins were in-gel reduced, alkylated and digested overnight with trypsin, AspN or chymotrypsin. Extracted peptides were analyzed by nanoliquid chromatography hyphenated to tandem mass spectrometry (nanoLC-MS/MS).

### Nanoliquid chromatography-Tandem Mass Spectrometry

NanoLC-MS/MS analyses were performed on a nanoACQUITY Ultra-Performance-LC system (UPLC, Waters, Milford, MA, USA) hyphenated to Q-TOF Impact HD (Bruker Daltonics, Bremen, Germany). Peptides were first trapped on a 0.18 mm × 20 mm, 5 μm Symmetry C18 precolumn (Waters) and then separated on an ACQUITY UPLC^®^ BEH130 C18 Column (Waters), 75 μm × 250 mm with 1.7 μm particle size. The solvent system consisted of 0.1% HCO_2_H in water (solvent A) and 0.1% HCO_2_H in ACN (solvent B). Trapping was performed for 3 min at 5 μL/min with 1% B. Elution was performed at a flow rate of 450 nl/min using a 5–45% (solvent B) over 60 min at 60 °C. The mass spectrometer was equipped with a CaptiveSpray source and a nanoBooster operating in positive mode. The source temperature was set at 150 °C while drying gas flow was at 3 L/min. The nano-electrospray voltage was optimized to −1300 V. External mass calibration of the TOF was achieved before each set of analyses using Tuning Mix (Agilent Technologies, Palo Alto, CA, USA) in the mass range of 322–2722 m/z. Mass correction was achieved by recalibration of acquired spectra to the applied lock mass hexakis (2,2,3,3,-tetrafluoropropoxy)phosphazine ([M+H]^+^=922.0098 m/z). For tandem MS experiments (CID), the system was operated with fixed cycle time of 3 s in the range of 150–2200 m/z. MS/MS scan speed was monitored in function of precursor intensity from 4 to 25 Hz. Ions were excluded after acquisition of one MS/MS spectra and the exclusion was released after 1 min. The complete system was fully controlled by Hystar 3.2 (Bruker Daltonics, Bremen, Germany).

### MS Data analysis

Mass data collected during nanoLC-MS/MS analyses were processed, converted into “.mgf” files with DataAnalysis 4.0 (Bruker Daltonics) and interpreted using MASCOT 2.4.1 (Matrix Science, London, UK) running on a local server. Searches were performed without any molecular weight or isoelectric point restrictions against an in-house generated protein database composed of protein sequences of *Plasmodium falciparum* PF3D7 (PlamoDB database, at September 24^th^ 2014) and of *Homo sapiens* (SwissProt, at September 24^th^ 2014). Known contaminant proteins such as human keratins and trypsin were added to the database. All proteins were concatenated with reversed copies of all sequences (51558 entries). A first round search was performed using full enzyme specificity, carbamidomethylation of cysteine was set as fixed modification, oxidation of methionine and protein N-terminal acetylation were set as variable modifications. Mascot results files were loaded into Proline software (Proline Studio Release 1.0). All spectra leading to an identification exceeding a minimum set threshold (Mascot Ion Score > 25, peptide length > 6 amino acids) and having a pretty rank (as defined by Mascot) equal to 1 were kept. Resulting spectra were then filtered to obtain a protein false discovery rate of less than 1%. This first round search enabled the identification of internal peptides and Nt-acetyl peptides in position 1 or 2 (after methionine excision). A second round search was performed to identify Nt-acetyl peptides in position >2 and semi-specific internal peptides. The Recover module of MSDA^37^ was used to create a subset “mgf” files by removing all identified spectra from the first round search, spectra with no peak above the m/z ratio of the 1+ charged state precursor ion and spectra which did not include at least six peaks higher than 2 times the intensity of the background noise. The second round search was performed using enzyme semi-specificity (only one end of a peptide needs to match the cleavage specificity) and as previously described. Mascot results files were loaded into Proline software and validated as previously described. Finally, a third round search was performed to identify free protein termini labelled by TMPP reagents. The Recover module was used a second time to create a second subset “mgf” files by removing identified spectra from the second round search and all spectra with a 1+ charged state precursor. The third round search was performed using Mascot algorithm with the following settings: enzyme semi-specificity, carbamidomethylation as fixed modification, light (+572.18 Da) and heavy (+581.21 Da) TMPP derivatization on any peptide N-terminal amino acid, side chain derivatization of lysine and tyrosine by light and heavy TMPP, oxidation of methionine were set as variable modifications. Mascot results files were loaded into Proline software and results were validated as described by Vaca Jacome *et al.*^28^ with a minimum Mascot ion score fixed at 15.

### Immunofluorescence assays and live cell imaging

Cells for immunofluorescence assays were fixed in 4% paraformaldehyde/0.00075% gluteraldehyde for 30 minutes at 37 °C as previously described^38^,^39^. Fixed cells were permeabilised with PBS/0.1% Triton X-100 for 10 minutes in the presence of 125 mM glycine, and quenching was performed using PBS/125 mM glycine. Blocking was achieved in PBS/3% BSA for 1 hour at room temperature. All antibodies were diluted in PBS/3% BSA. Primary antibodies: rabbit anti-PTEX150, anti-KAHRP (kind gift of B. Crabb 1:500), rabbit anti-SBP1 (BR5, a kind gift of Catherine Braun-Breton). Secondary antibody: anti-rabbit Cy3 (1:2000, DAKO). Hoechst 33258 (Molecular probes) was used in a final concentration of 50 ng/ml for fixed parasites and 10 μg/ml for live parasites, respectively. Images were acquired on Carl Zeiss Axio Observer inverse epifluorescence microscope and processed with ImageJ64 (version 1.46r, available at http://rsb.info.nih.gov/ij).

### Treatment with brefeldin A (BFA)

Highly synchronized parasites at early ring stage were cultured for 16 hr in the presence of 5 μg/ml Brefeldin A (Sigma) from a 4 mg/ml stock dissolved in ethanol before live cell imaging.

## Additional Information

**How to cite this article**: Rhiel, M. *et al.* Trafficking of the exported *P. falciparum* chaperone PfHsp70x. *Sci. Rep.* **6**, 36174; doi: 10.1038/srep36174 (2016).

**Publisher’s note:** Springer Nature remains neutral with regard to jurisdictional claims in published maps and institutional affiliations.

## Supplementary Material

Supplementary Information

## Figures and Tables

**Figure 1:**
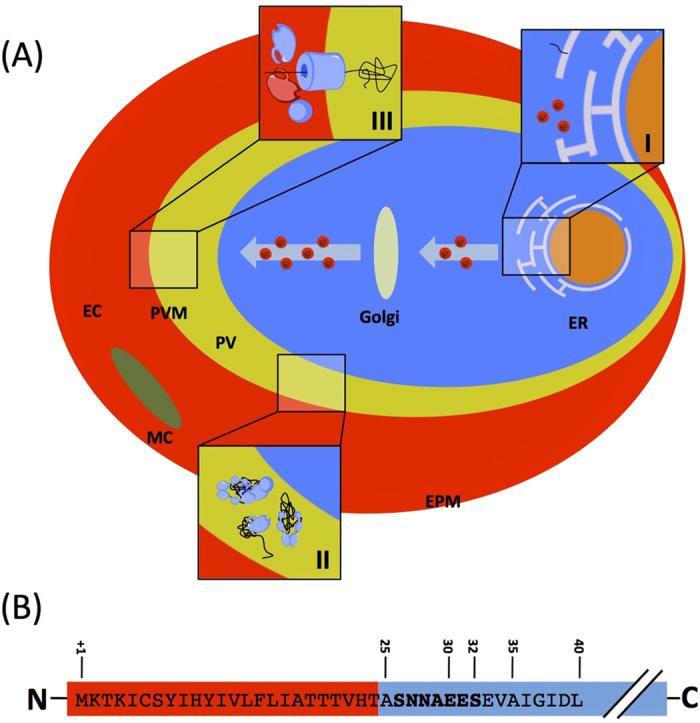
(**A**) General model of protein export in the *Plasmodium falciparum system*. Proteins enter the secretory pathway at the ER, directed by an N-terminal secretory signal sequence or an internal hydrophobic segment (I). Proteins continue along the secretory pathway and are released into the lumen of the parasitophorous vacuole where they undergo unfolding, possibly facilitated by molecular chaperones (II). Following unfolding, these proteins are believed to then pass through a membrane bound translocon before emerging into the erythrocyte cytosol where they then refold and are carried to various localisations (III). ER, endoplasmic reticulum; PV, lumen of the parasitophorous vacuole; PVM, parasitophorous vacuolar membrane; EC, erythrocyte cytosol; MC, Maurer’s cleft; EPM, erythrocyte plasma membrane. (**B**) Structure of the N-terminal region of PfHsp70x. Red indicates predicted signal peptide. Numbers refer to amino acid from the N-terminus. The PfHsp70x export motif is shown in bold.
