# Supplementary material for: Trafficking of the exported P. falciparum chaperone PfHsp70x
Source: Sci Rep. 2016 Nov 8;6:36174. doi: 10.1038/srep36174 (PMC5099922; doi:10.1038/srep36174)
Supplement: Supplementary Information [file srep36174-s1.pdf]

Trafficking of the exported *P. falciparum* chaperone PfHsp70x.

Manuel Rhiel[1, 2], Verena Bittl[1], Anke Tribensky[1], Sarah C Charnaud[3, 4], Maja Strecker[1], Sebastian Müller[1], Michael Lanzer[5], Cecilia Sanchez[5], Christine Schaeffer-Reiss[6], Benoit Westermann[6], Brendan S Crabb[3, 4, 7], Paul R Gilson[3, 4], Simone Külzer[1, 8], and Jude M Przyborski[1*]

Legends to Supplementary Material

Supplementary Figure S1. Trafficking of KAHRP and SBP1 is blocked upon inactivation of PTEX.

DIC, differential interference contrast; in merge and overlay blue, Hoechst (nuclear stain); green, GFP; red, specific antibody staining. Pictures are representative of at least 10 individual observations.

Supplementary Table S2. Oligonucleotides and cloning strategy

Trafficking of the exported *P. falciparum* chaperone PfHsp70x.

Manuel Rhiel[1, 2], Verena Bittl[1], Anke Tribensky[1], Sarah C Charnaud[3, 4], Maja Strecker[1], Sebastian Müller[1], Michael Lanzer[5], Cecilia Sanchez[5], Christine Schaeffer-Reiss[6], Benoit Westermann[6], Brendan S Crabb[3, 4, 7], Paul R Gilson[3, 4], Simone Külzer[1, 8], and Jude M Przyborski[1*]

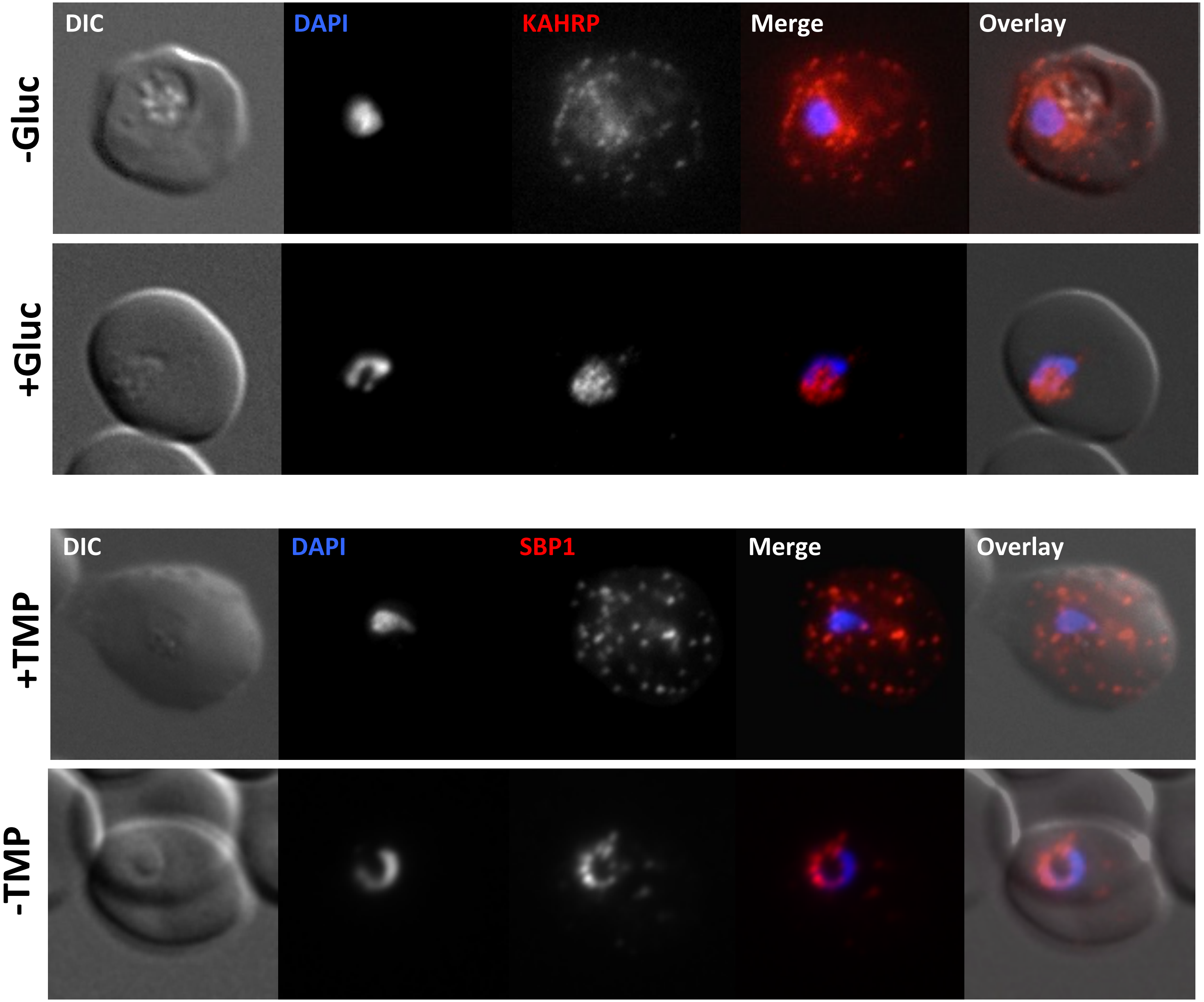

Supplementary Figure S1

Supplementary Table S2: Oligonucleotides used in this study

**Name**	**Sequence 5'→3'**
GFP1_10_oligo_F	CTAGGATGAGTAAAGGAGAAGAACTTTTCACTGGAGGTAC
GFP1_10_oligo_R	CTCCAGTGAAAAGTTCTTCTCCTTTACTCATC
Hsp70x_Xho_F	ggctcgagATGAAGACAAAAATTTGTAGTTATATTC
Hs70x_40_A_R	ggcctaggTAAATCAATACCAATTGCAACCTCTG
Hsp70x_FLP_AII_R	ggcctaggACTATTATTAGCTTCTTCACTTGCTGTATGTACTGTTGTTGTTGC
Hsp70x_E5/6D_AII_R	ggcctaggACTGTCGTCAGCATTATTACTTGCTGTATGTACTGTTGTTGTTGC
Hsp70x_E6A_AII_R	ggcctaggACTAGCTTCAGCATTATTACTTGCTGTATGTACTGTTGTTGTTGC
Hsp70x_E5/6K_AII_R	ggcctaggACTTTTTTTAGCATTATTACTTGCTGTATGTACTGTTGTTGTTGC
Hsp70x_N2/3A_AII_R	ggcctaggTGATTCTTCTGCAGCAGCACTTGCTGTATGTACTGTTGTTG
Hsp70x_SCR_AII_R	ggcctaggTTCACTTTCACTATTAGCATTTGCTGTATGTACTGTTGTTGTTGC
Hsp70x_S1A_AII_R	ggcctaggACTTTCTTCAGCATTATTAGCTGCTGTATGTACTGTTGTTGTTGC
Hsp70x_S7A_AII_R	ggcctaggAGCTTCTTCAGCATTATTACTTGCTGTATGTACTGTTGTTGTTGC
Sew_F_70x+STV	CAACAGTACATACACATTATGAAAATTATC
Sew_R_70x+STV	GATAATTTTCATAATGTGTATGTACTGTTG
stev^{A80rev}	gccctaggTTTCTTTATTGCGTCTTCG
Pfa660w_70x_A_R	ggctcgagATGGCAACCTTAAGGAAAAGCTATGTACC
Pfa660w_70x_X_F	ggcctaggTGATTCTTCTGCATTGTTACTTGCACAATTCACTGTAATTATTAAAAGGG
Flp_2NA_AII_R	ggcctaggACTGGCTGCAGCTTCTTCACTTGCTGTATGTACTGTTGTTGTTGC
Scr_2EA_AII_R	ggcctaggTGCACTAGCACTATTAGCATTTGCTGTATGTACTGTTGTTGTTGC

Restriction sites underlined; coding regions in capital letters

**Construct**	**Reverse Primer**	**Changed Export Motif**
S1A	Hsp70x_S1A_AII_R	**A**NNAEES
S7A	Hsp70x_S7A_AII_R	SNNAEE**A**
E6A	Hsp70x_E6A_AII_R	SNNAE**A**S
E5/6K	Hsp70x_E5/6K_AII_R	SNNA**KK**S
E5/6D	Hsp70x_E5/6D_AII_R	SNNA**DD**S
N2/3A	Hsp70x_N2/3A_AII_R	S**AA**AEES
SCR	Hsp70x_SCR_AII_R	S**ESENAN**
FLP	Hsp70x_FLP_AII_R	S**EEANN**S
FLP/2NA	Flp_2NA_AII_R	S**EEANN**S
SCR/2EA	Scr_2EA_AII_R	S**ESENAN**

Changes are highlighted in bold
